# Is Pneumonia an Essential Fever?

**Published:** 1889-02

**Authors:** B. F. Church

**Affiliations:** 104 West Seventh Street; Austin, Texas


DANIEL'S

### TEXAS MEDICAL JOURNAL.

A Representative Organ of the Medical Profession, and an Exponent of Rational Medicine; devoted to the Organization, Advancement and Elevation of the Profession in Texas.

PUBLISHED MONTHLY.—SUBSCRIPTION $2.00 A YEAR.

Vol. IV. AUSTIN, FEBRUARY, 1889. No. 8.

### ORIGINAL ARTICLES.

CONTRIBUTED EXCLUSIVELY TO THIS JOURNAL.

*The Articles in this Department are accepted and published with the understanding that we are not responsible for, nor do we indorse the views and opinions of the writers by so doing.*

### IS PNEUMONIA AN ESSENTIAL FEVER?

BY B. F. CHURCH, M. D., AUSTIN, TEXAS.

[Read before the Travis County Medical Society, January 10, 1889, and voted for publication in DANIEL'S TEXAS MEDICAL JOURNAL.]

AT the present time it seems to be the opinion of the majority of pathologists that the cause of pneumonia is to be sought in an infectious agent, which enters the lungs and there gives rise to the development of an inflammatory process. While pathological investigators have added much to strenghen this theory they have not at all times obtained the desired support of facts. Many seem so desirous of proving that pneumonia is an essential or infectious disease, makes it exceedingly probable that they may overlook other etiological factors of equal, or greater importance.

I do not intend, or deem it necessary, to even briefly review the history of the discovery of all the micro-organisms which have been supposed to be the cause of pneumonia, but, suffice to say whether it be the microbe found by Klebs in 1877, which he called the *monas pulmonale*, the capsule cocci of Freidlander, the spindle shaped encapsuled micro-cocci found by Frankel, or the spear formed micro-organism described by Weichselbium; the results have been the same, e. g., their faliure to invarably produce the characteristic desease of pneumonia after their inoculation into the lower animals. This Gordian knot as to whether the injection of these organisms into human lungs would cause pneumonia, may some time be untied by an enthusiastic student of medicine by auto-inoculation.

Freidlander's pneumonia coccus has been most spoken of in this country as a supposed cause of pneumonia. We will speak of these as the experiments with others have shown no better results.

Their special morphological characteristic is the presence of a gelatinous capsule which can be stained by certain aniline dyes. Flint in the last edition (6th) of his Practice of Medicine, says: "These bacteria have been found not only in the lungs after death, but also in the sputum during life, and in the sanguinolent fluid withdrawn by a hypodermic syringe from the hepatized lung during life. Injections of pure cultures into the lungs of mice cause lobular pneumonia, pleurisy, and invasion of the blood by the organisms. Pneumonia has been produced by the injection of the organisms into the lungs of dogs and Guinea pigs, but the effects are inconstant. Rabbits seem refractory to the inoculation of the organisms."

He further says: "The evidence that the capsule cocci of Freidlander are the infectious agents in lobar pneumonia cannot be considered conclusive. Among the objections to accepting them as the cause of pneumonia may be mentioned their inconstancy, the presence of other bacteria in pneumonia, and the possibility of producing similar results in animals by the inoculation of different kinds of bacteria. Bacteria, morphologically identical with the so called cocci of pneumonia, have been found in the sputum in the absence of pneumonia, Further observations, and experience are necessary in order to determiue the real significance of the pneumonic cocci.''

The researches of none of the investigators have brought to light a proof of the hypothesis that there is in the realm of bacteriology a specific cause for pneumonia. Yet, these scientists cling to the idea that such a pathogenic agent exists.

In the face of the chaotic information as regards this one point, would it not be better that we view a wider field and thereby make sure that other factors of equal, or greater importance are not overlooked, which may be more within our power to control?

The statement made by some that the traditional belief, which has existed since the time of Hippocrates, that cold may cause pneumonia, is unfounded; we need only to refer to statistics to prove their mistake. Knowing then that this disease is more prevalent during the winter months than the summer, even admitting some micro-organism to be its cause, it is certainly difficult to comprehend how, or why it selects the inhospitable winter season for its invasion, and be unusually energetic or swarm in greater numbers in the atmosphere during one of our ''northers'' in this state, when all reason teaches that these conditions are unfavorable for the generation, growth, or activity of any germ life, either animal, or vegetable.

One of the most extensive and comprehensive collections of statistics bearing upon this subject, is that reported by Dr. Henry Baker, of Lansing, Michigan, which can be found in the annual report of the Michigan State Board of Health. for the year 1886. He presents statistics of over 30,000 weekly reports of sickness, and 150,000 observations of atmospheric temperatures. His observations extend over several years, and include the statistics kept during the late war, representing nearly 50,000 cases of pneumonia, as well as statistics of 114,000 deaths in London from this disease.

In all of the reports (which are represented by disgrams) the author shows that the sickness curves foı pneumonia and bronchitis, follow very closely the curve for temperature.

His tables and diagrams for cloudiness and relative humidity of the atmosphere do not show curves closely related to the curve for pneumonia. Though a much closes relation exists between absolute humidity and pneumonia than is shown for the relative humidity.*

Next studying the tables exhibiting the relation of pneumonia in Michigan to the atmospheric pressure by months, (for a period of eight years), does not show a very close relation. The diagram however, showing the relation of pneumonia to the average daily range of atmospheric pressure there is a close connection, the greater the daily range of pressure the more pneumonia and *vice versa.*

It is interesting also to note, from observation in Michigan, the curve for atmospheric ozone by months is nearly the same as the curve for pneumonia.

With the above evidence it now remains to explain their action. In doing so, will make special reference to the theory advanced by Dr. Baker, of the accumulation of the non-volatile chlorides in the air cells of the lungs, with their pathological importance, which he so beautifully illustrates.

First, we will consider the effects of cold, dry air:

According to Prof. Guyot's tables, (of the Smithsonian) a cubic foot of air saturated with vapor of water, at zero, F: contains ½ gr.; at freezing point, (32 degrees F:) 2 grs.; at 70 degrees 8 grs., and at 98 degrees 18.69 grs.

Air, then is dry in proportion to its coldness. Kirk in his Physiology, says: "The air which is exhaled from the lungs leaves the body at about the temperature of the blood, and is always saturated with moisture."

It is estimated by different pysiologists that we daily exhale from our lungs and air passages from 11 to 16 ounces of water. Assuming 18 respirations per minute of 20 cubic inches of air

---

* By "absolute humidity" of air, is meant the amount of vapor of water in each cubic foot of air. While "relative humidity" is the per cent. of saturation of the air with vapor. 100 per cent. being its entire saturation. The capacity of air to contain vapor differs with each degree of temperature, therefore, statements of the relative humidity convey no knowledge of the actual quantity of vapor in the air unless the temperature is also stated.

each, and knowing its temperature, (bearing in mind the usual relation of the temperature to the actual quantity of water), we can approximately calculate the excess of moisture exhaled over that inhaled. Dr. Baker's tables show in Michigan for an average of eight years an excess of 8 ounces daily in the month of July, and 11 ounces daily in January.

The fluid which passes from the blood vessels into the air passages and air cells is not pure water, as is proved by analysis.

Prof. Flint says that "nasal mucus (the bronchial and pulmonary are quite similar) contains 5.09 to 5.69 parts per 1000 of sodium and potassium." The quantity of chlorides is about 50 per cent. greater than is contained in the blood, and nearly 100 per cent. more than is in the perspiration as it transudes. There is reason to think that, as the fluid normally exudes into the air passages it does not contain this large amount of chlorides, but, is left there as a deposit, and other conditions being equal, stands in direct proportion to the rapidity of the evaporation.

Dr. Baker formulates these facts in the following terse manner: "In passing from the blood vessels into the air cells and bronchial tubes the water takes with it the salts of the blood, perhaps especially the chloride of sodium; this salt does not readily pass out from the lungs with the vapor of water, and although normally, on account of endosmosis and action of the lymphatic system, no accumulation occurs, yet, under certain conditions, when the air inhaled is unusually cold and dry, and evaporation and transudation of the saline fluid is consequently excessively rapid, this salt accumalates in the air cells and bronchial tubes."

It has long been known that the chlorides were greatly diminished or entirely absent from the urine of persons sick with pneumonia during the stage of hepatization. This condition though is observed in other diseases.

The researches of Drs. Reatenbacker, Beale, and others, have proved that simultaneously with the disappearance of the chlorides from the urine in pneumonia, they appear in greater abundance in the lungs and sputa, and return to the urine as resolution begins. With these facts showing the great tendency of the chlorides to the lungs, it is easy to imagine how an "abortive"

case of pneumonia may occur. Supposing a minimum amount of salt in the body, and during an attack no more is taken in, a time will come when the blood is too poor in chlorides to give up more; the disease will be "self limited" unless the irritating matter in the air cells is of sufficient quantity to keep up the inflamation. If this is true, it may explain why pneumonia is so common in some of the Southern states, especially among the negroes where salted bacon and other salted meat is used so much for food.

As brought out by Dr. Baker's report, common salt in the system seems to favor two of the most important pathological conditions, which are found in pneumonia; that is, hyperinosis, and the fibrinous deposit in the lungs.

The hyperinosis, or increase of fibrin in the blood, is proved by the "buffy coat" on blood drawn from persons sick with pneumonia. Fibrin is practically oxidized albumen. Among substances which favor oxidation in the body, according to M. Albert Robin, common salt stands second. (Alcohol being first.)

As regards the accumulation of the exudate in the air cells, (having explained how the salts collected there by breathing unusually dry air), will quote from Dalton's Physiology on the law of osmosis. After speaking of the endosmosis of pure water through the shell membrane of a fowl's egg, he says:

"But a substance like albumen, which will not pass out by exosmosis toward pure water, may transudê a membrane which is in contract with common salt. This has been shown with the shell membrane of a fowl's egg, which, if immersed in a watery solution, containing 3 or 4 per cent. of sodium chloride, will allow the escape of a small proportion of albumen. If a mixed solution ot albumen and salt be placed in a dialysing apparatus, at first the salt alone will pass outward, leaving the albumen behind, but after the exterior liquid becomes perceptibly saline, the albumen also begins to transude in an appreciable quantity."

It seems probable that the characteristic exudate of pneumonia is brought about, or at least, favored by the long continued demand upon the air cells for water, which occurs through the inhalation of dry air; this extraordinary evaporation of water, leaving in the air cells an unusual quantity of the salts of the blood serum, which not only acts as an irritant, favoring the passage of the white blood corpuscles, but also favors the exudation of the albuminous constitutents of the blood serum.

Now that we have this albuminous fluid in the air cells, it is next in order to explain how it is changed into fibrin, for that it is a fibrinous deposit all agree. Referring to the tables of statistics just quoted, it is seen that the curve for the rise and fall of the atmospheric ozone closely follow the curve for the rise and fall of pneumonia, not so closely though (in a reverse order) as the temperature follows pneumonia.

As ozone is a powerful oxidizing agent, as is also shown of sodium chloride, and fibrin is chemically, oxidized albumen, these conditions are sufficient for one plausible explanation, at least.

Besides this effect of ozone upon the effused resum, it possibly acts as an irritant to the tubes and air cells, for it has been shown by Dr. Day, of England, (Dr. Baker's paper) that dogs caused to inhale artificially prepared ozone, (if prolonged), would produce pneumonia. It may also cause an excess of fibrin in the blood by its oxidizing properties.

As the tables show that sickness does not follow the rise and fall of atmospheric ozone as closely as it does the temperature, ozone is evidently not the controlling cause of pneumonia, but is doubtless a very important factor.

So far, the indirect effects of cold air only have been considered. Its direct effect should not be overlooked. After noticing the flush upon one's cheeks when entering a warm room from the cold outside air, it is not diffcult to imagine such a condition to take place in the highly organized and delicately constructed tissues of the lungs. The cold air first acts as a chemical irritant, paralysing the vaso-motor nerves of the arterioles, allowing them to dilate, constituting congestion, followed by the pathological law of exudation, emigration, and proliferation, which is inflammation itself. The danger of the development of pneumonia after the operation of tracheotomy or laryngotomy is well known. It is caused not only by the entrance of extraneous matter into the lungs, but from the direct effect of the cold, dry air not having been warmed and moistened by its passage through the nose, pharynx and larynx.

If the evidence which has been briefly reviewed in this paper be accepted; first, that the inhalation of unusually dry air has the effect to increase the transudation of fluid into the air cells and bronchial tubes, to maintain their moist condition; and second, by the rapid evaporation of this fluid an unusual quantity of salts (especially chloride of sodium) is deposited there; and third, by the law of osmosis, this accumulation of salts, together with the rapid evaporation of the water, favors the exudation of the albuminous constituents of the blood serum, also by its local irritating effect promotes the emigration of leucocytes into the air cells; and fourth, that the oxidizing power of these salts take a part in the transformation of this albuminous substance into fibrin, then there is no logical reason for doubting that warm, dry air may cause pneumonia.

It would naturally appear from this reasoning of the important place occupied by common salt in the causation of the bronchitis or pneumonia, that we should greatly lessen its consumption. This is not desirable; a "happy medium" should be sought.

All physiologists agree that a deficiency of sodium chloride in the food has an unfavorable influence on the general process of nutrition. Furthermore, normally, "this salt constitutes nearly 40 per cent. of the inorganic substances of the blood plasma, and protects the red corpuscles of the blood from solution."—(Dalton and Flint.)

The statistics referred to do not state the kind of pneumonia, whether croupous, or catarrhal. It is logical to suppose the causes enumerated to act in both forms.

The same agents may cause pharyngitis, laryngitis, or bronchitis, depending upon the locality of the air passages which supplied the extra moisture exhaled, and the abnormal heat and exudation which occurs after the affection of these parts may act as a preventive to pneumonia by rendering the air more suitable for its reception into the bronchioles and air cells, or, on the other hand, as catarrhal or lobular pneumonia is a secondary affection, the inflamation, when situated in the small bronchi, may extend by continuity to the adjacent air cells producing this type of the disease.

It is unnecessary to state that the science of bacteriology now stands above controversy, and that certain micro-organisms is the specific cause of many ills no one can refute, but, from the evidence before us it is highly probable, that, if any microbe has an influence in the causation of pneumonia it supplements and does not supplant other causes. The micro-organisms found in the lungs during hepitazation may be salutary, for they cannot be differentiated from those found in the mouths of healthy persons.

Pasteur has shown, that, of seventeen varieties of organisms found in the bucal secretion of persons in health, ten of this number when added to fibrin dissolved it completely. As the exudation of lobar or croupous pneumonia is not gotten rid of by expectoration, is not this fact not quite significant?

104 West Seventh Street.

